# Effects of Meteorological Parameters and PM_10_ on the Incidence of Hand, Foot, and Mouth Disease in Children in China

**DOI:** 10.3390/ijerph13050481

**Published:** 2016-05-10

**Authors:** Ruixue Huang, Guolin Bian, Tianfeng He, Lv Chen, Guozhang Xu

**Affiliations:** 1 Department of Occupational and Environmental Health, Xiangya School of Public Health, Central South University, Changsha 410078, China; huangruixue@csu.edu.cn; 2 Ningbo Center for Disease Control and Prevention, Ningbo 315010, China; biangl@nbcdc.org.cn (G.B.); hetf@nbcdc.org.cn (T.H.)

**Keywords:** distributed lag nonlinear model, hand, foot, and mouth disease, children

## Abstract

Hand, foot, and mouth disease (HFMD) is a globally-prevalent infectious disease. However, few data are available on prevention measures for HFMD. The purpose of this investigation was to evaluate the impacts of temperature, humidity, and air pollution, particularly levels of particulate matter with an aerodynamic diameter 10 micrometers (PM_10_), on the incidence of HFMD in a city in Eastern China. Daily morbidity, meteorological, and air pollution data for Ningbo City were collected for the period from January 2012 to December 2014. A total of 86,695 HFMD cases were enrolled in this study. We used a distributed lag nonlinear model (DLNM) with Poisson distribution to analyze the nonlinear lag effects of daily mean temperature, daily humidity, and found significant relationships with the incidence of HFMD; in contrast, PM_10_ level showed no relationship to the incidence of HFMD. Our findings will facilitate the development of effective preventive measures and early forecasting of HFMD outbreaks.

## 1. Introduction

Hand, foot, and mouth disease (HFMD) is a common human syndrome caused by a highly contagious intestinal virus. HFMD is typically characterized by a mild fever, followed by a rash of flat discolored spots and bumps that may blister, involving the skin of the hands, feet, mouth, and occasionally the buttocks and genitalia [1,2,3]. Infants and children are particularly susceptible to HFMD [4]. 

Coxsackievirus A16 is the most common cause of HFMD [4], followed by enterovirus 71 (EV-71) [4]. Numerous other strains of coxsackievirus and enterovirus can also be responsible for the disease. The viruses that cause HFMD are spread through direct contact with the mucus, saliva, or feces of an infected person. During the past few decades, large outbreaks of HFMD have been reported worldwide [5]. HFMD was first recognized in China in the 1980s. On 3 May 2008, Chinese health authorities reported a major outbreak of EV71 in Fuyang City and other localities in Anhui, Zhejiang, and Guangdong provinces. In 2008, an outbreak in China, beginning in March in Fuyang, Anhui, led to 25,000 infections and 42 deaths by May 13 [6]. According to the World Health Organization, HFMD infected 1,520,274 people in China, with 431 deaths reported through the end of July 2012. No antiviral treatment or vaccine is currently available for HFMD; thus, the establishment of an early protective barrier is important.

Human evolution involves adaptation to the environment, which improves survival. Human genes are affected by changes in ambient temperature and air pollution. Due to their smaller bodies and ongoing development, children are affected to a greater extent by increases in temperature and air pollution [7,8]. Much epidemiologic evidence suggests that HFMD is sensitive to changes in climate. Temperature has been linked to the incidence of HFMD; however, the findings are inconsistent [9,10,11]. Hii *et al.* reported a positive relationship between temperature and the incidence of HFMD. If the temperature is above 32 °C, the incidence of HFMD increased by 36% per each degree [12]. However, another study conducted in China revealed a negative relationship between HFMD and temperature [13]. These studies suggested that other factors, such as air pollutants, contribute to the influence of temperature on the incidence of HFMD. Such pollution comprises suspended particulates, including PM_10_—particulate matter with an aerodynamic diameter 10 micrometer. Since PM_10_ can enter the human respiratory system, and even penetrate lung cells, thereby entering the blood circulation, it damages human health, especially in children. The relationship between air pollution and respiratory diseases of children, particularly asthma, is well established [14]. The authors of two multicenter studies conducted in Europe [15] and Australia [16] reported an overall non-significant association between major air pollutants and asthma-related emergency room visits, with the exception of nitrogen dioxide (NO_2_) which is a reddish-brown gas above 70 °F with a pungent, acrid odor, becomes a yellowish-brown liquid below 70 °F, and converts to the colorless dinitrogen tetroxide below 15 °F. However, the impact of exposure to major air pollutants on the incidence of HFMD has not been fully determined. 

**Figure 1:**
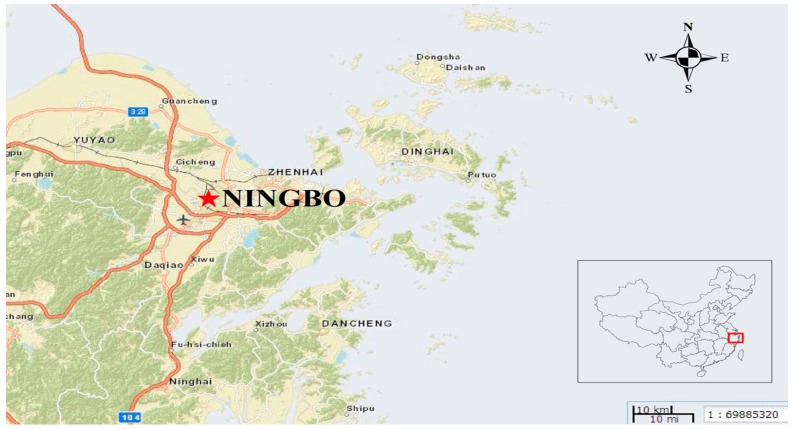
Map of Ningbo City, Zhejiang Province, China ([17]).

The purpose of this research was to evaluate the effects of temperature and PM_10_ on the incidence of HFMD in Ningbo, a coastal city in China. We performed a time-series analysis of the relationship between daily HFMD incidence and meteorological and air pollutant panels from Jane 2012 to December 2014 in Ningbo (120°55′ E, 28°51′ N), Zhejiang Province, China. Figure 1 represents the geographical situation. The city is located near the sea and comprises six districts, with an area of 9816 km^2^ and a population of 7.811 million in 2014. This city has a subtropical monsoon climate with a high temperature, a large amount of precipitation in summer, and a warm, wet winter. The city has experienced a disproportionately high incidence of HFMD; a number of severe HFMD cases in children resulted in neurogenic pulmonary edema and several deaths. We hypothesize that PM_10_ is readily suspended in the atmosphere, blocking sunlight, which may contribute to virus reproduction and affect the ambient temperature. A study of the influences of temperature and PM_10_ on the incidence of HFMD would facilitate elucidation of features of the disease and early. Furthermore, the findings will give scientific evidence to enable the government to formulate and implement appropriate strategies or policies to reduce the incidence of HFMD.

The distributed lag nonlinear model (DLNM) has been used to analyze non-linear effects and to quantify the effects of individual lags. We used this method to assess the influences of temperature and PM_10_ and to identify and model the log relative risks of each lag [7].

## 2. Methods

### 2.1. Data Collection

All data from HFMD cases recorded between January 2012 and December 2014 were obtained from the Ningbo Center for Disease Control and Prevention, China. The standard for the clinical diagnosis of HFMD was based on the 2009 guidelines of the Chinese Ministry of Health [18]. Daily data on mean temperature, relative humidity, wind velocity, PM_10_, NO_2_, and sulfur dioxide (SO_2_), the chemical compound with the formula SO_2_, at standard atmosphere, it is a toxic gas with a pungent, irritating smell, were obtained from the China Meteorological Data Sharing Service System. Our research team considered pediatric HFMD cases, as HFMD is a greater threat to the health of children than to that of adults.

### 2.2. Data Analysis

Due to the existence of lag effects, the outcome for a given day may be affected by the situation in the few previous days; thus, the effects of temperature and PM_10_ on the incidence of HFMD were evaluated using a DLNM model. The effects of long-term and seasonal trends were controlled for by means of a smoothing spline function. The model is shown below:
***Y***_t_~poisson (*μ*t)log(*μ*_t_) = α + ns(T_t,1_,df) + ns(NO_2_,df) + *φ* atmospheric pressure + ns(PM_10 t,1_,df) + σ windvelocity + *γ*SO_2_ + ns(RH_t,1_,df) + *j*DOW_t_, + *к*Strata_t_
where t represents study days, Yt is the number of daily HFMD cases on day t (t = 1, 2, 3, 4 …, 1095), *μ*_t_ relative risk for incidence of HFMD, α is the intercept, and l is the number of lag days.

According to the cross-basis function in the DLNM, T_t,l_, RH_t,l_, and ns(RH_t,l_, df) are temperature and associated humidity, respectively. df are the degrees of freedom; after controlling for long-term and seasonal trends, the df was 4 [19,20]. In 2001, Beaga adjusted the model and put forward the idea that imposed some restrictions on the lag distribution. It can use some appropriate function transformation, such as utilizing the idea of strata to assume that a certain section lag had the same fixed effect [21]. Strata, in this model, is a categorical parameter of the year and calendar month because the calendar month and the year are time we use trata to control for long-term trends and seasonality. *к* is a vector of coefficients. DOWt is the week parameter and *j* is a vector of coefficients. Atmospheric pressure and wind velocity were removed from the model, as they were not related significantly to the incidence of HFMD. A maximum lag of 13 days was used to explore the potential associations of lag with temperature, relative humidity, and PM_10_ in our model, to eliminate the role of the natural incubation period (3–7 days) [22]. Spline knots were placed at equal intervals in the temperature, humidity, and PM_10_ ranges and in the log scale of lag using the default setting of the DLNM. To explore the association of hand, foot, and mouth disease with air pollutants original air pollution data (interval scale) were be used. We used the R software (version 3.1.0) (Peter Dalgaard, Frederiksberg, Denmark) and the “dlnm” package to conduct a stratified analysis [22]. Spearman’s correlation tests were two sided. *p*-values < 0.05 were taken as indicative of statistical significance. All risk estimates were presented with corresponding 95% confidence intervals. Sensitivity analyses were performed by changing the maximum lag for DLNM from 13 days to 20 days and the df for weather variables from 4 to 7. This study was a re-analysis of data that were collected by Ningbo Center for Disease Control. The research proposal was approved by the Institutional Review Boards (IRB) of Ningbo Center for Disease Control (201601). Data analyses were de-identified.

## 3. Results

### Study Characteristics

A total of 86,695 HFMD cases were reported in Ningbo City between 2012 and 2014. Demographic characteristics are shown in Table 1.

Table 1 and Table 2 show the parameters of HFMD cases recorded in 2012–2014. Children aged <15 years were the principal victims in the outbreaks, as these comprised 99.63% of all reported cases. Of the patients with HFMD, 51,989 (59.97%) were male and 34,706 (40.03%) were female. Table 2 shows the meteorological conditions and air pollutant levels in Ningbo for 2012–2014. The mean temperature was 17.64 °C, the mean relative humidity was 73.17%, the mean wind velocity was 11.88 m/s, the mean PM_10_ concentration was 90.69 mg/m^3^, the mean NO_2_ level was 46.19 mg/m^3^, and the mean SO_2_ level was 19.45 mg/m^3^.

**Figure 2:**
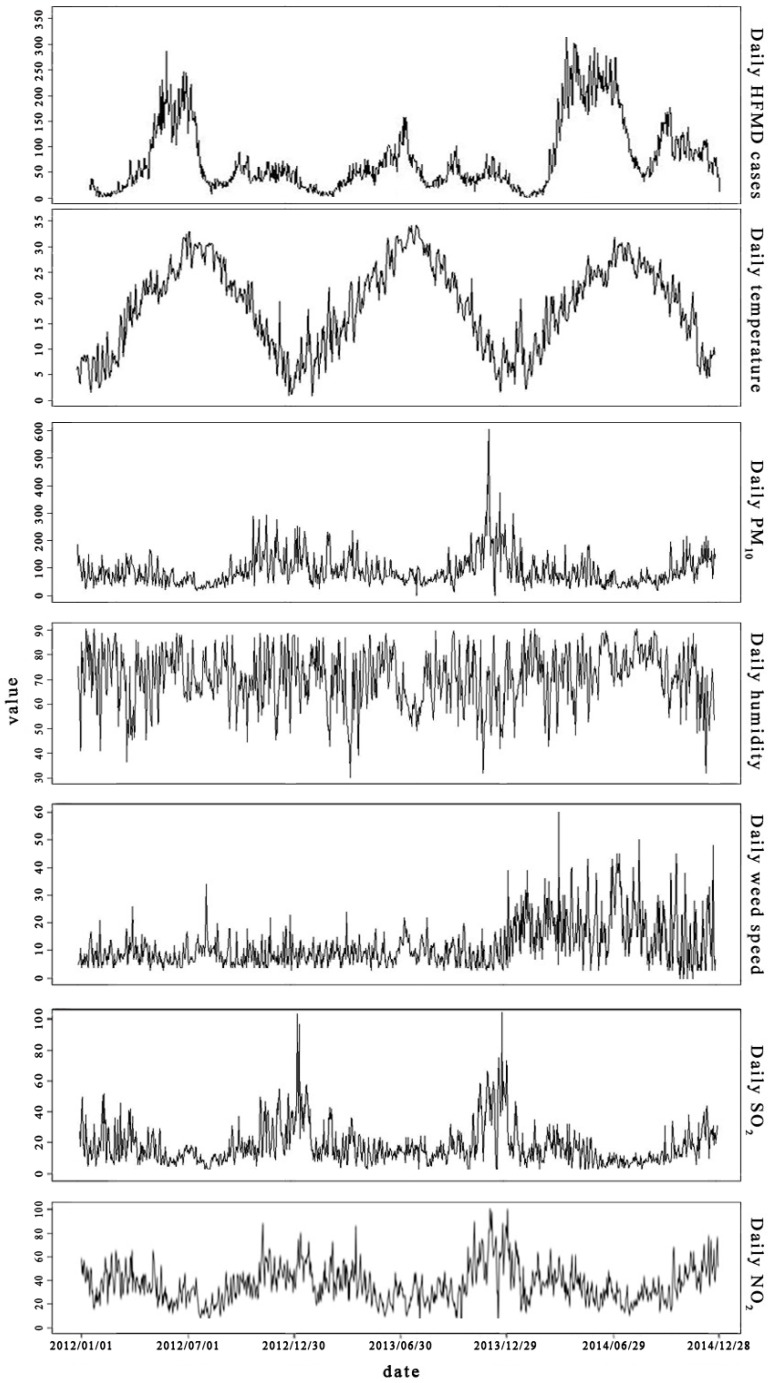
Daily distribution of HFMD incidence and mean temperature, humidity, wind speed, NO_2_ level, SO_2_ level, and PM_10_ level in Ningbo.

Figure 2 shows daily distribution of HFMD incidence and mean temperature, humidity, wind speed, NO_2_ level, SO_2_ level and PM_10_ level in Ningbo. The incidence of HFMD in Ningbo exhibited significant seasonal variation.

The incidence peaks of HFMD disease concentrated in June and July, respectively, in accordance with the temperature peaks in July, however, the daily level of PM_10_ were similar except for a few days with high levels of PM_10_, and the daily humidity and daily wind speed levels are similar without significant peaks during the period of data collection.

## 4. Discussion

HFMD infects mainly infants and children and can result in serious complications, such as pneumonia and even death. As no vaccine is available, prevention of HFMD is important. Temperature influences the incidence of HFMD. However, few studies have examined the effects of temperature and PM_10_ level on the incidence of HFMD. We assessed the relationships of temperature and PM_10_ with the incidence of HFMD in Ningbo City. To our knowledge, this study is the first to assess the relationship between PM_10_ level and the incidence of HFMD in this area of China. We applied a DLNM to daily data, and evaluated nonlinear associations and cumulative risks according to temperature and PM_10_ levels for different numbers of lag days.

First, we analyzed the effect of temperature on the incidence of HFMD in Ningbo, and found a positive relationship, which was similar to reports from Singapore, Hong Kong, Guangzhou, Qingdao, and Jinan [7,9]. However, the use of different models in these studies may have led to variation in the results. We detected a nonlinear association between temperature and the incidence of HFMD. Thus, had the data been subjected to a simple linear regression, the result would have been different. A DLNM was used in this study because it provides a detailed representation of nonlinear exposure–response relationships and avoids co-linearity issues of co-linearity among lagging exposure variables compared with other models, such as the generalized linear model or the generalized additive model [23]. Therefore, the DLNM has been used frequently to analyze relationships between meteorological parameters and various diseases, such as asthma.

The use of appropriate temperature indicators is important when assessing the association between temperature and the incidence of HFMD. Maximum, minimum, and mean temperatures are the indicators used most frequently in previous studies. The daily mean temperature is considered to be the most accurate indicator because it represents an entire 24-h period, whereas maximum and minimum temperatures represent only points in time [24,25]. In our opinion, temporal scale is an issue that should be considered in the use of meteorological variables, as the frequent use of aggregated variables could influence the results. In our research, the detailed temporal scale provided more-accurate information on the incidence of HFMD, followed by the impending temperature; in contrast, previous studies have used the weekly or monthly incidence of HFMD, which is insufficiently sensitive and specific for use in early warning systems [26]. The effects of changes in temperature differ among viruses in terms of their growth, multiplication, and spread. Socioeconomic and medical standards, as well as disease prevention and control policies, can influence the outcomes of assessments of temperature–disease associations.

Epidemiological data indicate that temperature has an important influence on the airborne transmission of infectious diseases. However, pathogens, host populations, and environmental factors can influence each other, resulting in a complex web of temperature–disease interactions. Virus infectivity is influenced by temperature—virus multiplication increases with temperature in a certain range, which facilitates its spread throughout a population [27]. Furthermore, outdoor activity levels increase in summer due to the pleasant temperatures. Therefore, human behavior is related to temperature and the increasing behaviors may result the people assembly and increase the HFMD virus infection chance. A previous study showed that population of cities near the sea are more trend to engage in doing activities than the population of inland areas [7]. A high frequency of outdoor activities in public places could increase the risk of infection. Ningbo is densely populated, and many tourists travel to this city for sightseeing each year, particularly in summer. A few HFMD cases may result in an outbreak, or even an epidemic. This situation could explain the marked effect of high temperature in Ningbo. Another research based on lab experiments showed that enteroviruses could live in good condition in wet environments; therefore, a greater number of precipitation days and higher humidity may exacerbate HFMD epidemics in the cities near the sea.

It is interesting to note that dairy humidity was positively associated with HFMD incidence, it is observed that RR peaked at a lag of six days, which is combined with the earlier finding [28]. Humidity changes should be considered important in the development of HFMD control programs.

Ambient air pollution may also influence the incidence of HFMD in Ningbo City. Studies of relationships between PM_10_, a major air pollutant, and various diseases have accumulated. According to previous reports, early-life exposure to ambient air pollution is associated with childhood asthma [28]. The relationship between PM_10_ and cardiovascular morbidity has also been investigated [29].

**Figure 3:**
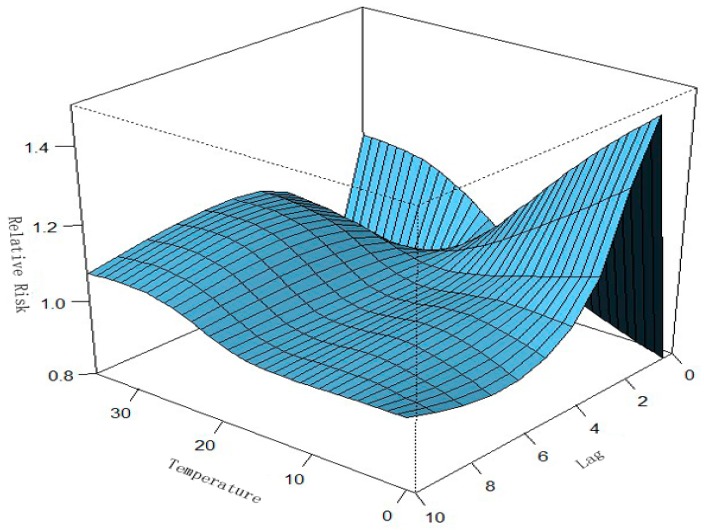
Relationships among relative risk (RR), temperature, and the number of lag days in Ningbo. After adjusting for relative humidity, wind velocity, wind direction, holidays, seasonal trend, and long-term trends, a three-dimensional plot of RR, temperature, and lag days (*n* = 10) was generated. Temperature had a nonlinear effect on the incidence of HFMD. We found that the association of temperature with HFMD may have a different lag pattern. For example, the extreme high temperature (31 °C) had a high RR for HFMD cases on the current and fifth lag days.

**Figure 4:**
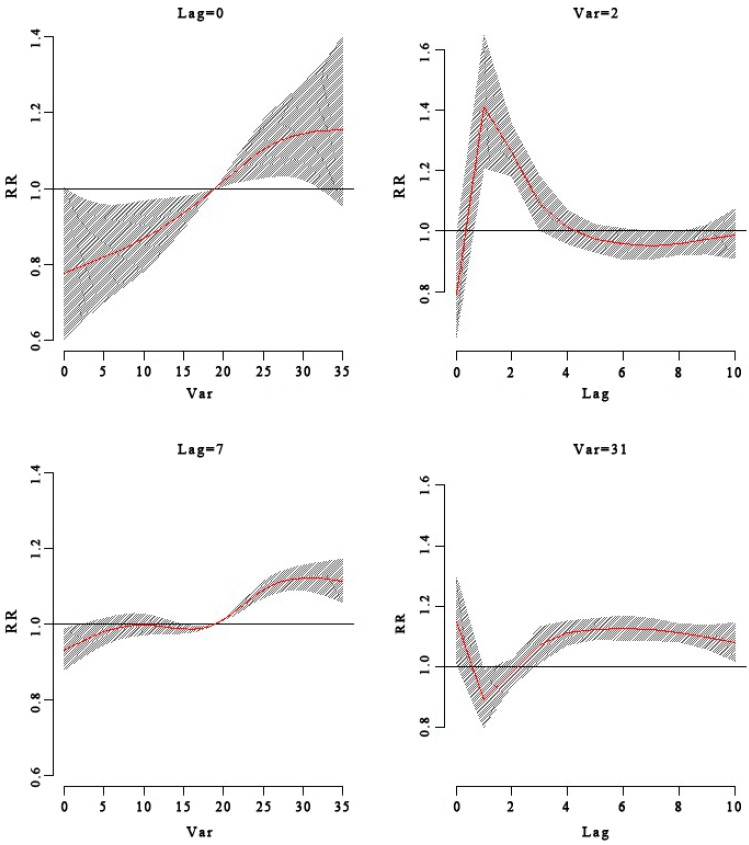
The RR of HFMD at a specific lag day (0 day,7 day) and at different temperatures (2 °C, 31 °C). At a temperature of 31 °C, there was high RR for HFMD cases. The relationship between RR of HFMD and temperatures had a lag effect. At a temperature of 31 °C, the RR of HFMD had a high result at current day and then decreased for two days and then turned to an increase until the fourth lag day; however, the low temperature (2 °C) had the minimal RR on the current day and had the maximum RR at the second lag day. It considered that the relationship between high temperatures displayed earlier and lasted a longer time than the relationship between low temperatures and HFMD cases.

**Figure 5:**
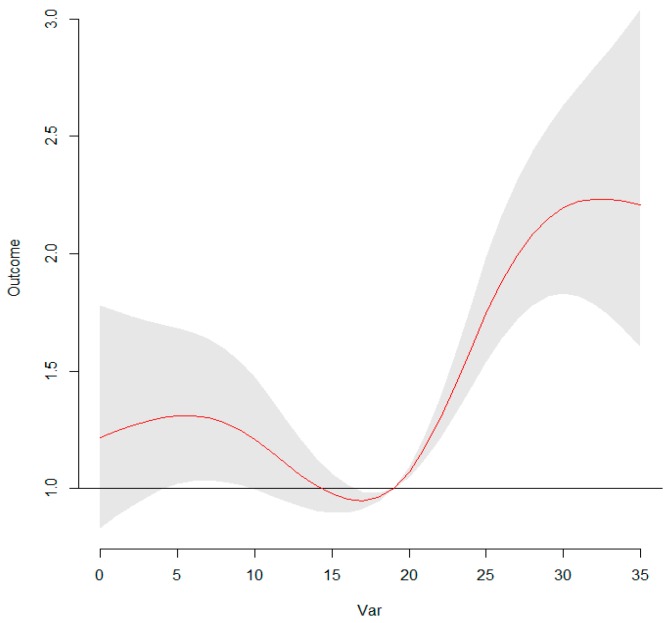
The overall RR of temperature (°C) for total HFMD cases. The RRs increased with the increment of temperature and it reached the peak at 31 °C.

**Figure 6:**
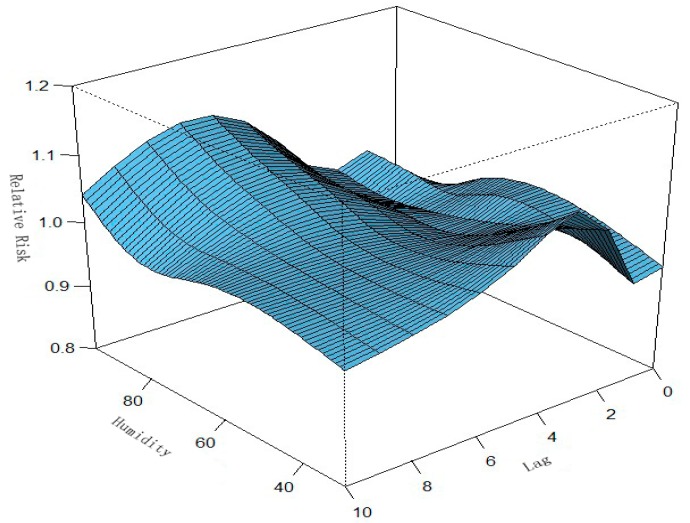
Relationships among relative risk (RR), humidity, and number of lag days in Ningbo. A three-dimensional plot of RR, humidity, and lag days (*n* = 10) was generated. Humidity had a nonlinear effect on the incidence of HFMD. We found that the association of humidity with HFMD may have a different lag pattern. For example, the extreme high humidity (90%) had a high RR for HFMD cases on the seventh lag day.

**Figure 7:**
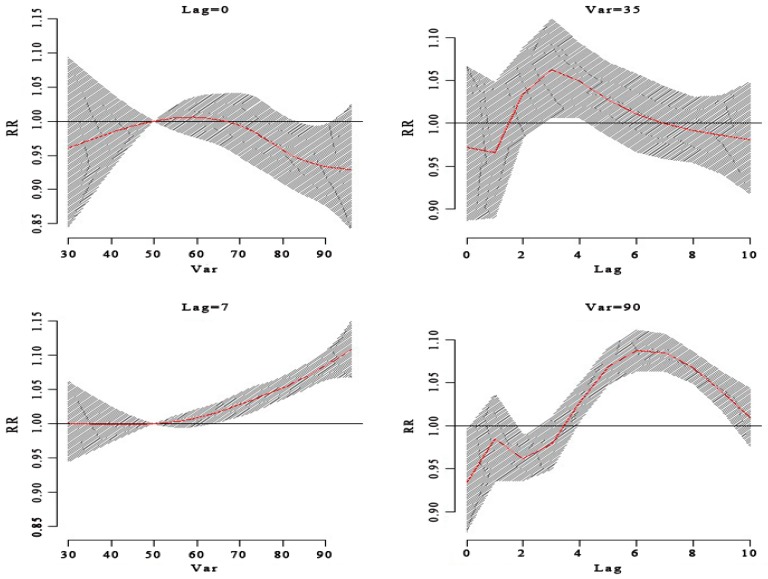
The RR of HFMD by humidity at a specific lag day (0 day, 7 day) and different humidity (35%, 90%). It shows at 90% humidity that there was high RR for HFMD cases. The relationship between the RR of HFMD and humidity had a lag effect. At 90% humidity, the RR of HFMD had a low result on the current day and then turned to an increase until the seventh lag day. When the humidity was 35%, it had a high RR of HFMD cases at the third lag day; however, when the humidity was 90%, the high RR of HFMD cases displayed at the sixth lag day.

**Figure 8:**
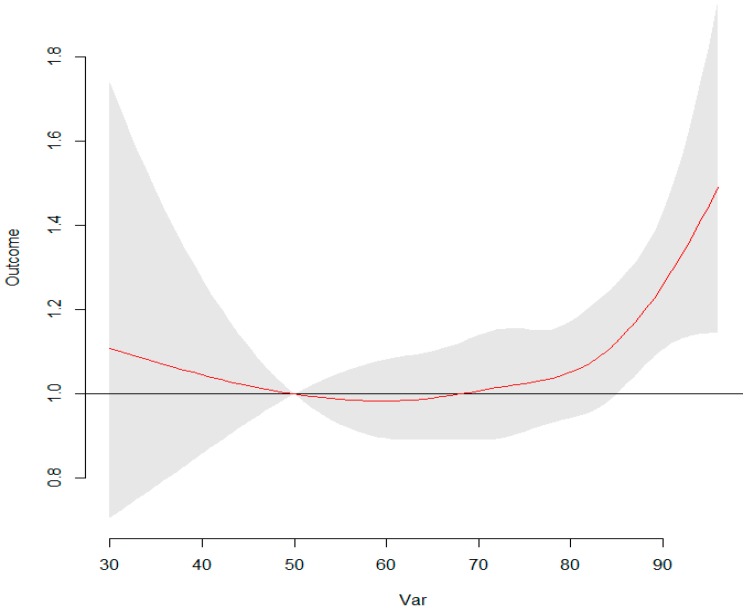
The overall RR of humidity for total HFMD cases. The RRs increased with the increment of humidity and it arrived the peak at humidity (90%).

**Figure 9:**
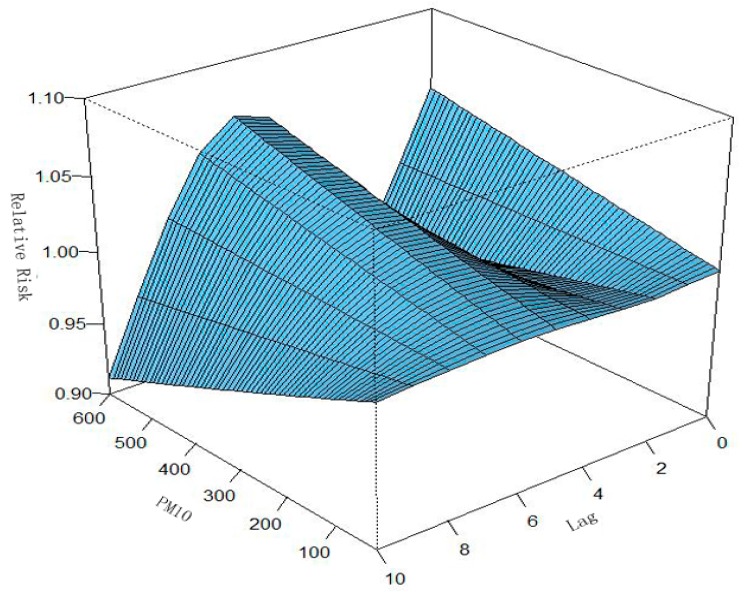
Relationships among RR, PM_10_, and lag days in Ningbo after adjusting for relative humidity, wind velocity, wind direction, holidays, seasonal trend, and long-term trend. A three-dimensional plot of RR, PM_10_, and lag days (*n* = 10) was generated.

**Figure 10:**
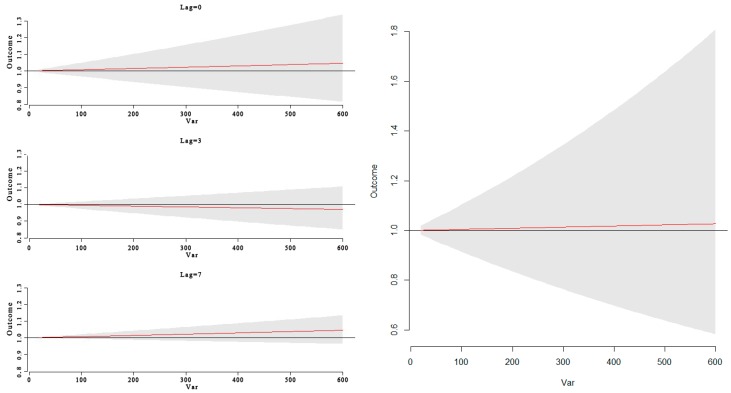
The RR of HFMD cases by PM_10_ at a specific lag days (0 day, 7 day) and the overall relative risk of HFMD according to PM10 level during a 10-day period. It shows no significant correlation was found between the RR of HFMD cases and PM_10_.

To our knowledge, no previous report has examined the relationship between PM_10_ level and the incidence of HFMD. We found that the incidence of HFMD was not significantly associated with the PM_10_ level, but strongly associated with temperature and humidity according to the analysis results from Figure 3, Figure 4, Figure 5, Figure 6, Figure 7, Figure 8, Figure 9 and Figure 10. These results may be due to decreased levels of outdoor activity when the ambient air pollution level is high. Industrial activity may also have increased the level of air pollution, as we observed no significant seasonal peak in PM_10_ concentration from 2012 to 2014. Until now, sufficient evidence infers a relationship between air pollution and respiratory diseases. A study in Australia found that geogenic PM_10_ exposure increases inflammation, impairs lung function, and increases viral load, exacerbating the response to respiratory viral infection [30]. Another study reported PM_10_ seems to play an important role in the transmission of Q fever from infected animals to humans [31]. PM has been demonstrated to induce the formation of an excessive amount of reactive oxygen species in respiratory systems of experimental animals, leading to tissue inflammation and cell death [32]. In addition, there is evidence that ambient PM in a polluted urban environment could induce oxidative stress in humans [33]. It is believed that more severe respiratory symptoms including cough, phlegm, and dyspnea can be caused by air pollution. If people lived in an environment with air pollution including PM_10_ then the whole respiratory system is constantly under attack. It can be inferred that the more serious damage of the respiratory system is, the more easy it is for HFMD to infect and damage the body. Children that live in developing countries are the most vulnerable population in terms of total deaths attributable to indoor and outdoor air pollution because of the children being in their growth stage, physical and psychological, are not mature, and their organs are more sensitive to air pollution. Protective measures to ensure children avoiding HFMD disease should be enacted in China urgently. The association between ambient air pollution and the incidence of HFMD warrants further investigation.

Several limitations of this study should be acknowledged. First, some major risk factors for HFMD disease including season, suburban, ultraviolet (UV) radiation, childhood aggregation, socio-economic status [34], as well as environmental factors such as temperature, PM_10_, and PM_2.5_ should be considered together to adjust them in the models. We adjusted only the meteorological parameters and PM_10_ in the model, which may have led to outcome bias, thus, the next step is to improve the statistical methods. Second, we used meteorological and air pollution data from a fixed site, instead of individual exposure data, which may have led to measurement errors. Third, this study focused on a single city; therefore, the results can be applied to cities with similar meteorological and air pollution parameters, but not necessarily to those with different meteorological and air pollution parameters.

## 5. Conclusions

In conclusion, our study revealed a nonlinear relationship between temperature, humidity, and the incidence of HFMD in children in a coastal city in China. Our study first demonstrated that there is no significantly relationship between PM_10_ and HFMD incidence. Although the complexity of HFMD cannot be fully explained by weather factors, our results provide new quantitative evidence indicating the influence of weather factors on HFMD infections at a finer spatial-temporal scale. Further studies in other cities are required to confirm these findings and verify the effect of ambient air pollution on the incidence of HFMD.

## Figures and Tables

**Table 1:** Basic demographic characteristics of HFMD cases in Ningbo, 2012–2014.

Basic Demographic Characteristics	Ningbo
Total Cases	86,695
Age	
0–1 years	7103 (8.19%)
1–3 years	44,270 (51.06%)
3–6 years	30,492 (35.17%)
6–15 years	4525 (5.21%)
15–50 years	298 (0.34%)
>50 years	7 (0.03%)
Sex	
Male	51,989 (59.97%)
Female	34,706 (40.03%)

**Table 2:** Meteorological and air pollutant data for Ningbo, 2012–2014.

Index	Mean ± SD	Min.	Median	Max.
Temp (°C)	17.64 ± 8.82	−0.8	18.95	34.4
Humidity	73.17 ± 12.34	28	74	95
Atmospheric pressure	1015.51 ± 8.67	986.61	1015.59	1037.55
Wind velocity	11.88 ± 8.02	3	10	60
PM_10_	90.69 ± 56.91	13	74	605
NO_2_	46.19 ± 20.73	7	44	132
SO_2_	19.45 ± 13.23	4	16	108
